# Investigating the L-Glu-NMDA receptor-H_2_S-NMDA receptor pathway that regulates gastric function in rats’ nucleus ambiguus

**DOI:** 10.3389/fphar.2024.1389873

**Published:** 2024-05-01

**Authors:** Hongzhao Sun, Chenyu Li, Yuan Shi, Yiya Wang, Jinjin Li, Linkun Fan, Yan Yu, Xiaofeng Ji, Xiaoting Gao, Keyuan Hou, Yuxue Li

**Affiliations:** College of Life Science, Qi Lu Normal University, Jinan, China

**Keywords:** nucleus ambiguus, hydrogen sulfide, L-glutamate, gastric motility, gastric acid secretion

## Abstract

**Background:**

In previous investigations, we explored the regulation of gastric function by hydrogen sulfide (H_2_S) and L-glutamate (L-Glu) injections in the nucleus ambiguus (NA). We also determined that both H_2_S and L-Glu have roles to play in the physiological activities of the body, and that NA is an important nucleus for receiving visceral sensations. The purpose of this study was to explore the potential pathway link between L-Glu and H_2_S, resulting in the regulation of gastric function.

**Methods:**

Physiological saline (PS), L-glutamate (L-Glu, 2 nmol), NaHS (2 nmol), D-2-amino-5-phopho-novalerate (D-AP5, 2 nmol) + L-Glu (2 nmol), aminooxyacetic acid (AOAA, 2 nmol) + L-Glu (2 nmol), D-AP5 (2 nmol) + NaHS (2 nmol) were injected into the NA. A balloon was inserted into the stomach to observe gastric pressure and for recording the changes of gastric smooth muscle contraction curve. The gastric fluid was collected by esophageal perfusion and for recording the change of gastric pH value.

**Results:**

Injecting L-Glu in NA was found to significantly inhibit gastric motility and promote gastric acid secretion in rats (*p* < 0.01). On the other hand, injecting the PS, pre-injection N-methyl-D-aspartate (NMDA) receptor blocker D-AP5, cystathionine beta-synthase (CBS) inhibitor AOAA and re-injection L-Glu did not result in significant changes (*p* > 0.05). The same injection NaHS significantly inhibit gastric motility and promote gastric acid secretion in rats (*p* < 0.01), but is eliminated by injection D-AP5 (*p* > 0.05).

**Conclusion:**

The results indicate that both exogenous L-Glu and H_2_S injected in NA regulate gastric motility and gastric acid secretion through NMDA receptors. This suggests that NA has an L-Glu-NMDA receptor-CBS-H_2_S pathway that regulates gastric function.

## 1 Introduction

The number of patients with gastric dysfunction is progressively increasing. The occurrence of gastric ulcers is usually accompanied by hyperactive gastric function and increased secretion of gastric acidLy et al. (2015). As a major excitatory neurotransmitter in the central nervous system (CNS), glutamate plays a fundamental role in regulating physiological (memory and learning) and pathophysiological (stroke, epilepsy, and neurodegenerative diseases such as Alzheimer’s disease and Parkinson’s disease) conditionsMeldrum (2000); Filpa et al. (2016).

The altered gastric function is mainly attributed to the increased activity of the parasympathetic nerves that innervate the stomach. These nerves are derived from the dorsal motor nucleus of the vagus (DMV) and the nucleus ambiguus (NA) of the medulla oblongata. The NA is the final efferent pathway of the central parasympathetic nervous system that regulates gastrointestinal function and visceral activity. The excitatory neurons in the NA may be involved in gastric dysfunction that is induced by restraint water-immersion stress (RWIS). They may have various roles in the regulating gastric function Zhang et al. (2009).

L-Glu is an excitatory neurotransmitter that is widely present within the mammalian central nervous system and is closely associated with rapid excitatory synaptic transmission and the pathogenesis of certain neurological disorders. Gastrointestinal motility is regulated by various factors such as ghrelin, gastrin, and nitric oxide. Glutamate receptor-mediated gastrointestinal motility has been frequently reported in the past decades. However, reports on the significance of N-methyl-D-aspartate (NMDA) receptors in the pathogenesis of gastrointestinal motility disorders are limited and relatively rare. On the other hand, there has been a recent surge in the number of reports on cholinergic transmission associated with the vagus nerve that affects the regulation of gastrointestinal motility induced by NMDA receptor blockadeWatanabe et al. (2008). There are two categories of Glu receptors (GluRs), which are, ionotropic glutamate receptors (iGluRs) and metabotropic glutamate receptors (mGluRs). Previous experiments have shown that in the NA, L-Glu inhibits gastric motility in rats via the NMDA receptors Sun et al. (2014). L-Glu functions as a neurotransmitter in the nucleus tractus solitarius (NTS) to DMV connection. Glutamatergic neurons stimulate vagal efferent nerves in the DMV and play a key excitatory role in regulating gastrointestinal contraction. In fact, mice injected with L-Glu via IV can stimulate the contraction of gastric smooth muscles Horita et al. (2018).

Hydrogen sulfide (H_2_S) is now a well-recognized gas transmitter after nitric oxide (NO) and carbon monoxide (CO), and it is endogenously produced in the brain and other various human organ tissues Wang (2002). Many studies have revealed that H_2_S controls the progression of physiopathological conditions such as Parkinson’s disease Cao et al. (2018) and Alzheimer’s disease Eto et al. (2002). H_2_S is a small molecule gas that can easily cross various biological membranes and has a wide range of targets. It may exert its regulatory effects by affecting multiple signaling pathways Calvert et al. (2010). In the brain, the H_2_S produced by cystathionine beta-synthase (CBS) regulates the physiological aspects of cells via the NMDA receptors on glutamate Dawe et al. (2008). The physiological concentrations of H_2_S enhance the excitatory postsynaptic potentiation that is mediated by NMDA. Through the NMDA receptors, H_2_S can also dose-dependently ease hippocampal long-range memory enhancement in the CA1 region of the hippocampus Abe and Kimura (1996).

The current study was conducted to investigate the effects of the neuronal cytosolic stimulant L-Glu and exogenous H_2_S donor NaHS on gastric motility and gastric acid secretion in the NA, and to investigate whether the L-Glu-NMDA receptor-CBS-H_2_S pathway that regulates gastric function exists in NA.

## 2 Materials and methods

### 2.1 Experimental animal preparation

The animals used in this experiment were 270–320 g healthy male Wistar rats purchased from Jinan Pengyue Experimental Animal Breeding Co. Initially, the rats rats were first caged in a quiet animal room with controlled room temperature (22 ± 2)°C, natural rhythmic light, free access to food and water, and fed standard chow for a week after acclimatization. After that, the formal experiment was then conducted. The rats fasted for 24 h before the experiment and were fed water *ad libitum*. The Experimental Animal Ethics Committee of Qilu Normal University approved the study, and the project number is 14276-2022-066. All experiments complied with internationally accepted ethics and followed the guidelines by the International Association for the Study of Pain Zimmermann (1986).

### 2.2 Chemicals

NaHS, L-Glu, D-AP5, and potamine sky blue were purchased from Sigma-Aldrich (St. Louis, MO, United States). Concentrations of 0.02 mol/L were prepared for L-Glu, NAHS, AOAA, and D-AP5 solutions, and 2% Potamine sky blue solution was prepared for the identification of nucleosome stimulation sites. All reagents were prepared in 0.9% NaCl solution.

### 2.3 Experimental grouping

To investigate the presence of the L-Glu-NMDA receptor-CBS-H_2_S pathway in the NA that regulates gastric function, experiments were grouped as follows: 1) Effects of microinjecting L-Glu (0.1 *μ*L, 0.02 mol/L) on gastric motility (*n* = 6) and gastric acid secretion (*n* = 6) in the NA; 2) Effects of microinjecting PS (0.1 *μ*L, *n* = 6) on gastric motility (*n* = 6) and gastric acid secretion (*n* = 6) in the NA as a control group; 3) Effects of microinjecting D-AP5 (0.1 *μ*L, 0.02 mol/L) + L-Glu (0.1 *μ*L, 0.02 mol/L) on gastric motility (*n* = 6) and gastric acid secretion (*n* = 6) in the NA; 4) Effects of microinjecting AOAA (0.1 *μ*L, 0.02 mol/L) + L-Glu (0.1 *μ*L, 0.02 mol/L) on gastric motility (*n* = 6) and gastric acid secretion (*n* = 6) in the NA; 5) Effects of microinjecting NaHS (0.1 *μ*L, 0.02 mol/L) on gastric motility (*n* = 6) and gastric acid secretion (*n* = 6) in the NA; 6) Effects of microinjecting D-AP5 (0.1 *μ*L, 0.02 mol/L) + NaHS (0.1 *μ*L, 0.02 mol/L) on gastric motility (*n* = 6) and gastric acid secretion (*n* = 6) in the NA.

### 2.4 Rat brain stereotactic localization

Rats were anesthetized with chloral hydrate (approximately 400 mg/kg body weight) intraperitoneally prior to surgery. When the generalized muscles of the rats were relaxed, their corneal reflexes were blunted, and breathing was slow and uniform. The rats were ready for surgery.

The rats were fixed in dorsal position, and then the skin was cut along the mid-abdominal line under the sternal raphe to expose the muscular layer. Then, an incision was made in the abdominal wall along the abdominal white line to pull out the stomach. A small incision was made on the glandular stomach to clean the residue of the stomach, and a 5 mm diameter water sac was inserted into the pylorus of the gastric sinus. The abdominal wall opening was sutured in layers. The rats were kept warm with a heat lamp.

The rat head was fixed on a brain stereotaxic apparatus (Stoelting 68002, Shenzhen Ruiwode Company, China), and the animal was fixed according to George Paxinos and Charles Watson (6th edition) brain stereotaxic atlas of the rat, using the animal’s bilateral inner ear holes with the three points of the incisors, while adjusting the tooth rest. The height of the brackets was adjusted to be 3.3 to 0.4 mm below the level of the line connecting the two ear bars, with the anterior and posterior chimneys being at the same level.

The hair on the top of the rat’s skull was removed using hair clippers. The scalp was cut along the sagittal suture of the skull using ophthalmic clippers to expose the skull. The excess connective tissue around the skull was cut away, the skull’s surface was gently wiped using physiological saline until the fontanelle and herringbone suture were exposed. The positions of the anterior and posterior chimneys were determined. The 3D coordinates of the anterior chimney were recorded as the zero point, and the center point of NA on the coronal section (anterior chimney 12.7 mm backward along the median line, 9.6 mm deep) was drilled on the skull using a small bone drill, based on the coordinates of the coronal section in the atlas. A small hole with a diameter of about 2 mm was drilled in the skull using a bone drill. A glass microelectrode with a tip of about 40 ∼60 *μ*m was inserted into the brain tissue according to the coordinate depth value.

### 2.5 Recording gastric motility methods and microinjection of drugs

The balloon inserted into the rat stomach was connected to a pressure transducer and BL-420 (Biological Function Experimental System; Chengdu Taimeng Company, China) through a polyethylene plastic tube. The normal gastric motility curve was first recorded until it was regular. Then, 0.1 *μ*L of L-Glu, NaHS, PS, D-AP5 (or AOAA) were injected into the NA of the rats at a uniform rate within 1 min. Gastric motility was then recorded for 10 min after the injection. After the experiment, 0.1 *μ*L of 2% potamine sky blue was injected into the NA of rats at a uniform rate within 1 min, as a marker to identify the accuracy of the stimulation site. The transducer recording parameters were as follows: speed, 25 mm/min; sensitivity 0.5 mV/cm; filtering 10 Hz.

### 2.6 Recording gastric acid secretion

Gastric fluid was collected using the esophageal perfusion method. A 2.5 mm inner diameter polyethylene tube was inserted into the trachea at the neck to maintain smooth breathing. A polyethylene tube with an inner diameter of 2 mm was also inserted for esophageal intubation. Pyloric intubation was performed in the abdomen by cutting a small opening at the pylorus and duodenum junction where there are few blood vessels. Then, a polyethylene tube with an inner diameter of 3 mm was inserted into the stomach through the opening and a dry Petri dish was used to receive the gastric fluid. Perfusion was performed with 0.9% saline at 37°C, pH 7.4, at a rate of 2 mL/min. The gastric juice was first flushed for 15 min to remove the gastric residue, thereby stabilizing gastric acid secretion. The gastric juice was then collected every 10 min, and its pH was measured using a pH meter. It was collected three consecutive times as a control before drug injection. After stimulation, the gastric juice was collected three more times, consecutively. This was done to compare the gastric juice secretion before and after drug injection.

### 2.7 Heart perfusion

After the rat was sacrificed with an overdose of isoflurane, the heart was exposed by opening the thoracic cavity of the rat along the flush position of the sternal process. The aorta was exposed by gently removing the fatty tissue around the heart using surgical scissors. The needle of the infusion set was inserted from the left ventricle of the heart to the aorta, and the right ear of the rat was quickly cut open to facilitate blood flow until the fluid flowing from the right atrium was light yellow. The flow rate of the syringe was adjusted and the heart was perfused with 500 mL of 0.01 mol/L phosphate-buffered saline (PBS) and 500 mL of 4% 0.1 mol/L paraformaldehyde (PFA). The flow rate of PFA was reduced when twitching limbs or tail wagging was observed on the rat. After the completion of perfusion, the rat’s head was cut off to expose the skull and the brain was extracted, placed in a small wide-mouth flask containing 4% PFA and then fixed at 4°C for 24 h. The rat’s brain tissue was dehydrated using 30% sucrose solution for 48 h. Frozen coronal sections were made after the rat’s brain tissue had settled.

### 2.8 Microinjection sites histological identification

First, the slides were pretreated with chromium-vanadium gelatin, and then the blocks of rat tissue that had been fixed and dehydrated were removed, tail-end up, fixed on the carrier table of a frozen sectioning machine, and frozen at −16°C for half an hour. Then, 16-*μ*m-thick consecutive coronal sections were made. The sections were continuously adhered to the slides. Photographic observations were made using a microscope (Nikon Optiphot, Nikon, Shanghai, China) and a digital camera (Magnafire; Optronics, Goleta, CA, United States) that was connected to a computer. Based on the Wistar rat atlas, the location of the blue dots was determined under the microscope.

### 2.9 Data analysis and statistics

The mean amplitude and mean time of 5-min gastric contractions before and after microinjection, along with the mean value of the pH at three consecutive 10-min intervals before and after microinjection were counted for comparison, respectively. The average gastric motility index (A.G.M.I) was the product of the average amplitude of contraction waves (A.A.C.W) and the average duration of contraction waves (A.D.C.W). To determine the change in gastric motility before and after injection, the gastric motility inhibition rate was calculated as follows: Inhibition rate (%) = (pre-injection value - post-injection value) × 100%/pre-injection value. The rate at which gastric acid secretion was facilitated was calculated as follows: Facilitation rate (%) = (average of three pH values 10 min after injection - average of three pH values 10 min before injection) × 100%/average of three pH values 10 min after injection.

The results were analyzed using SPSS v25.0 (IBM SPSS Inc., Chicago, IL, United States).Use one-way ANOVA and Student’s t-test, followed by Student-Newman-Keuls test for *post hoc* testing. Multiple comparisons between groups were performed using the least significant difference (LSD) test. All data are shown as mean ± SEM. *p*-values <0.05 were considered statistically significant.

## 3 Results

### 3.1 NA injection site identification

**FIGURE 1:**
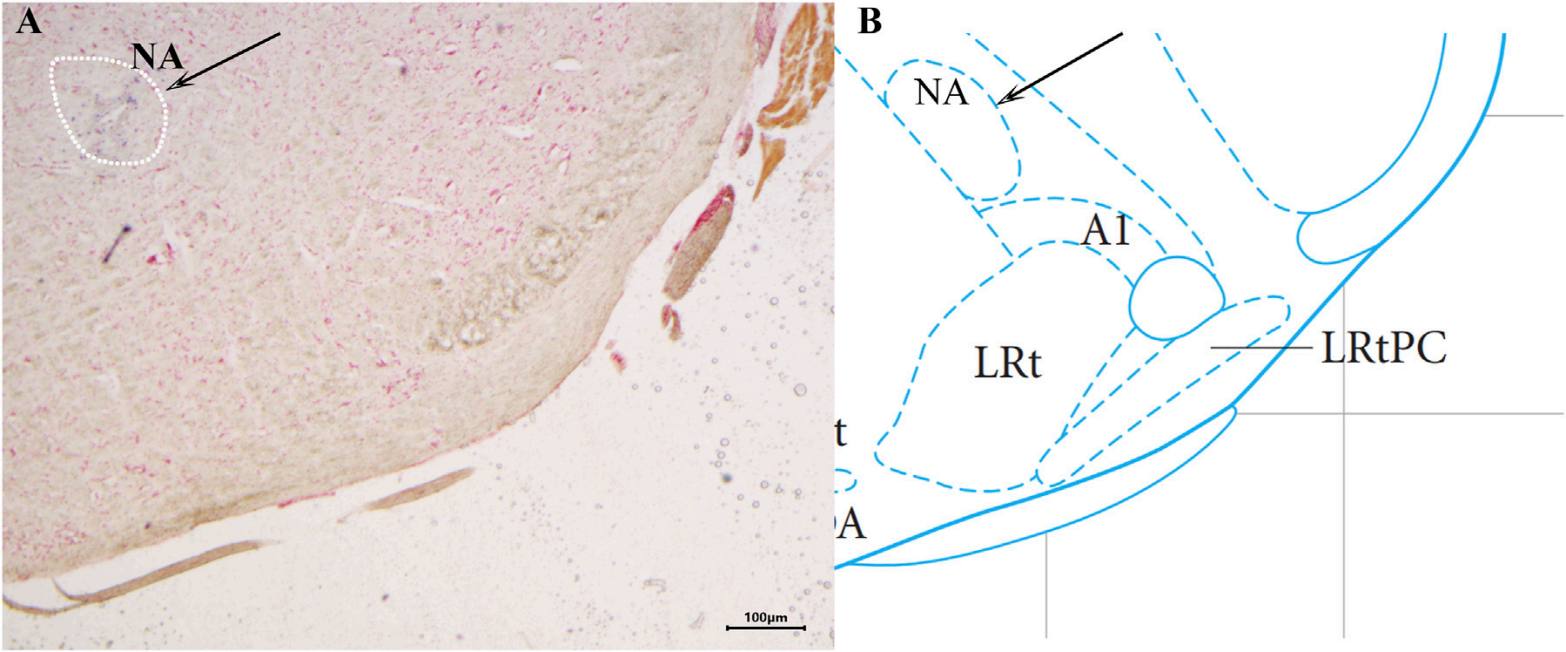
Histological identification of microinjection, the location of the nucleus ambiguus (NA) in the brain. ×40 **(A)** A brain section stained with neutral red. The blue dot represents injection into the NA. **(B)** The position of the NA in the brain atlas.

The brain slices were stained with neutral red and placed under a light microscope to determine the location of the injected blue spots against the atlas. Animals with the blue spot location at the NA (Figure 1) were taken for statistical analysis.

### 3.2 Injecting D-AP5 and AOAA into NA eliminates the inhibitory effect of 2 nmol L-Glu on gastric motility in rats

**FIGURE 2:**
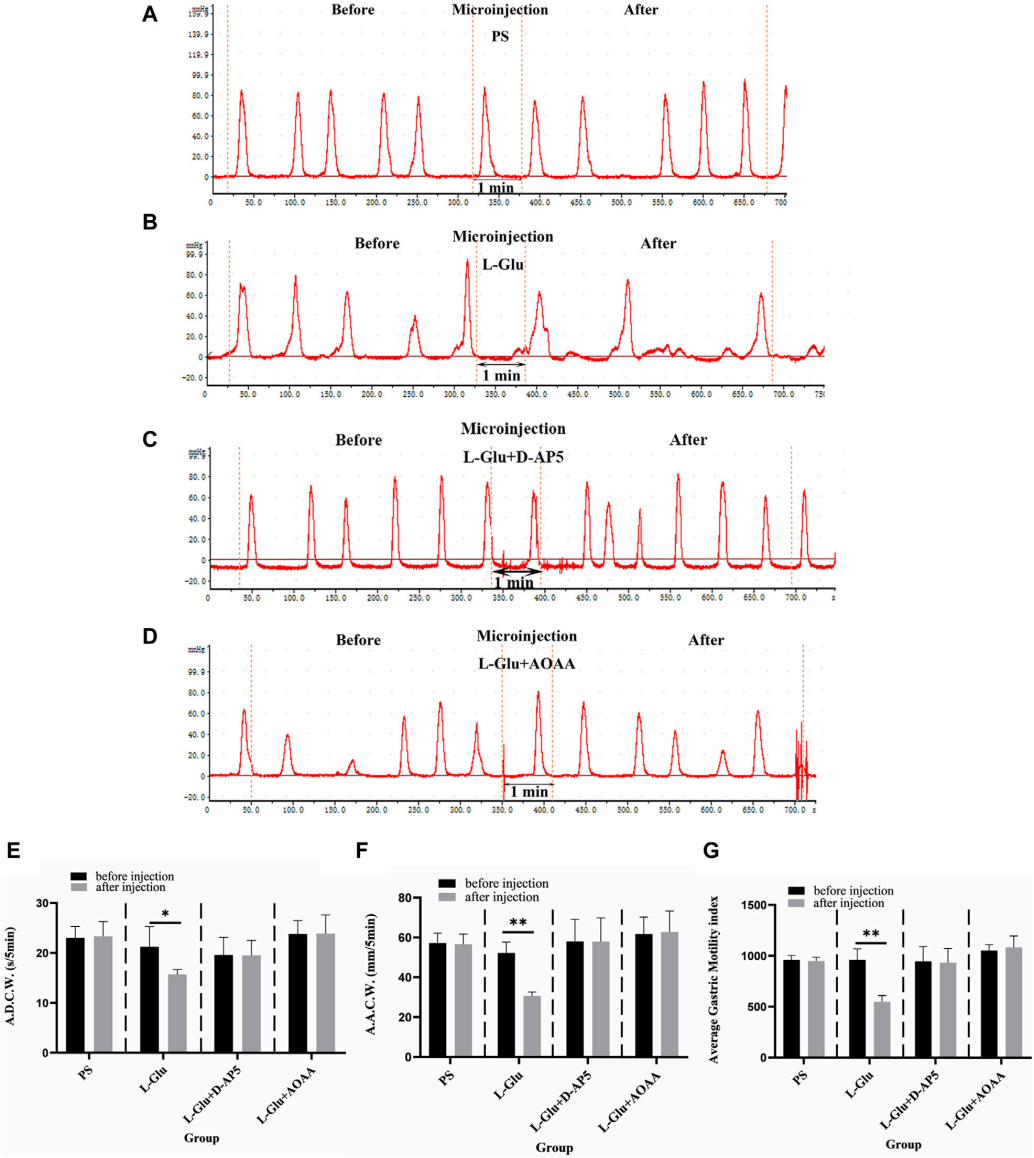
Effect of L-glutamate (L-Glu) microinjection in nucleus ambiguus (NA) on gastric motility in rats. **(A)** The gastric motility curve of physiological saline (PS) group, **(B)** The gastric motility curve of L-Glu group, **(C)** The gastric motility curve of D-2-amino-5-phopho-novalerate (D-AP5) + L-Glu group, and **(D)** The gastric motility curve of aminooxyacetic acid (AOAA) + L-Glu group **(E)** Average duration of contraction waves (A.D.C.W). data before and after microinjection of PS, L-Glu, D-AP5 + L-Glu and AOAA + L-Glu (*n* = 6). **(F)** Average amplitude of contraction waves (A.A.C.W) data before and after microinjection of PS, L-Glu, D-AP5 + L-Glu and AOAA + L-Glu (*n* = 6). **(G)** Average gastric motility index (A.G.M.I) data before and after microinjection of PS, L-Glu, D-AP5 + L-Glu and AOAA + L-Glu (*n* = 6). **p < 0.01,*p < 0.05, after microinjection compared with before microinjection.

After 2 nmol L-Glu was injected into the NA, the images for gastric motility 5 min after drug injection were compared with those that represented 5 min before drug injection. It was found that the A.D.C.W., A.A.C.W., and A.G.M.I. of gastric motility in rats were relatively inhibited after than before (Figure 2B). When physiological saline (PS), D-AP5 + L-Glu and AOAA + L-Glu into the NA, no significant changes were observed in all the indices of gastric motility after the injection compared with those that were recorded before (Figures 2A,C,D).

The data from the observations that were done 5 min before and after microinjection were also measured, analyzed, and compared. At the NA microinjection dose of 2 nmol L-Glu, the contraction wave of A.A.C.W reduced from 52.22 ± 2.27 mm 5 min^−1^ to 30.74 ± 0.77 mm 5 min^−1^ (*p* < 0.01); the contraction wave of A.D.C.W decreased from 21.27 ± 1.80 s 5 min^−1^ to 15.74 ± 0.42 s 5 min^−1^ (*p* < 0.05); and the A.G.M.I decreased from 962.03 ± 47.88 to 549.01 ± 27.63 (*p* < 0.01) (Figures 2E–G). After PS was injected into the NA as the control group, the A.A.C.W of the systolic wave went from 57.24 ± 2.18 mm 5 min^−1^ to 56.69 ± 2.24 mm 5 min^−1^, the A.D.C.W of systolic wave went from 23.04 ± 1.02 s 5 min^−1^ to 23.36 ± 1.31 s 5 min^−1^, and the A.G.M.I dropped from 960.10 ± 19.29 to 949.82 ± 16.70 (Figures 2E–G).

Microinjecting D-AP5 (an NMDA receptor blocker) followed by L-Glu in the NA eliminated the inhibitory effect of L-Glu on gastric motility. The data 5 min before and 5 min after microinjection were also measured, analyzed, and compared. The A.A.C.W of the contraction waves changed from 58.09 ± 4.96 mm 5 min^−1^ to 58.05 ± 5.26 mm 5 min^−1^; the A.D.C.W of contraction waves dropped from 19.63 ± 1.58 s 5 min^−1^ to 19.57 ± 1.33 s 5 min^−1^ after D-AP5 injection followed by L-Glu injection; and the A.G.M.I reduced from 948.41 ± 64.50 to 933.68 ± 62.52 (Figures 2E–G). These data suggest that L-Glu can regulate gastric motility through the NMDA receptors.

Microinjection with AOAA (a CBS inhibitor), followed by L-Glu in the NA, eliminated the inhibitory effect of L-Glu on gastric motility. The data 5 min before and 5 min after microinjection were measured, analyzed, and compared. The A.A.C.W of the systolic wave changed from 61.75 ± 3.82 mm 5 min^−1^ to 62.82 ± 4.71 mm 5 min^−1^; the A.D.C.W of the systolic wave increased from 23.84 ± 1.19 s 5 min^−1^ to 23.91 ± 1.68 s 5 min^−1^ after AOAA followed by L-Glu injection; and the A.G.M.I changed from 1054.94 ± 25.39 to 1086.13 ± 49.74 (Figures 2E–G). These data suggest that L-Glu can regulate gastric motility via the NMDA receptors, having passed through CBS neurons.

### 3.3 Injecting D-AP5 into NA eliminates the inhibitory effect of 2 nmol NaHS on gastric motility in rats

**FIGURE 3:**
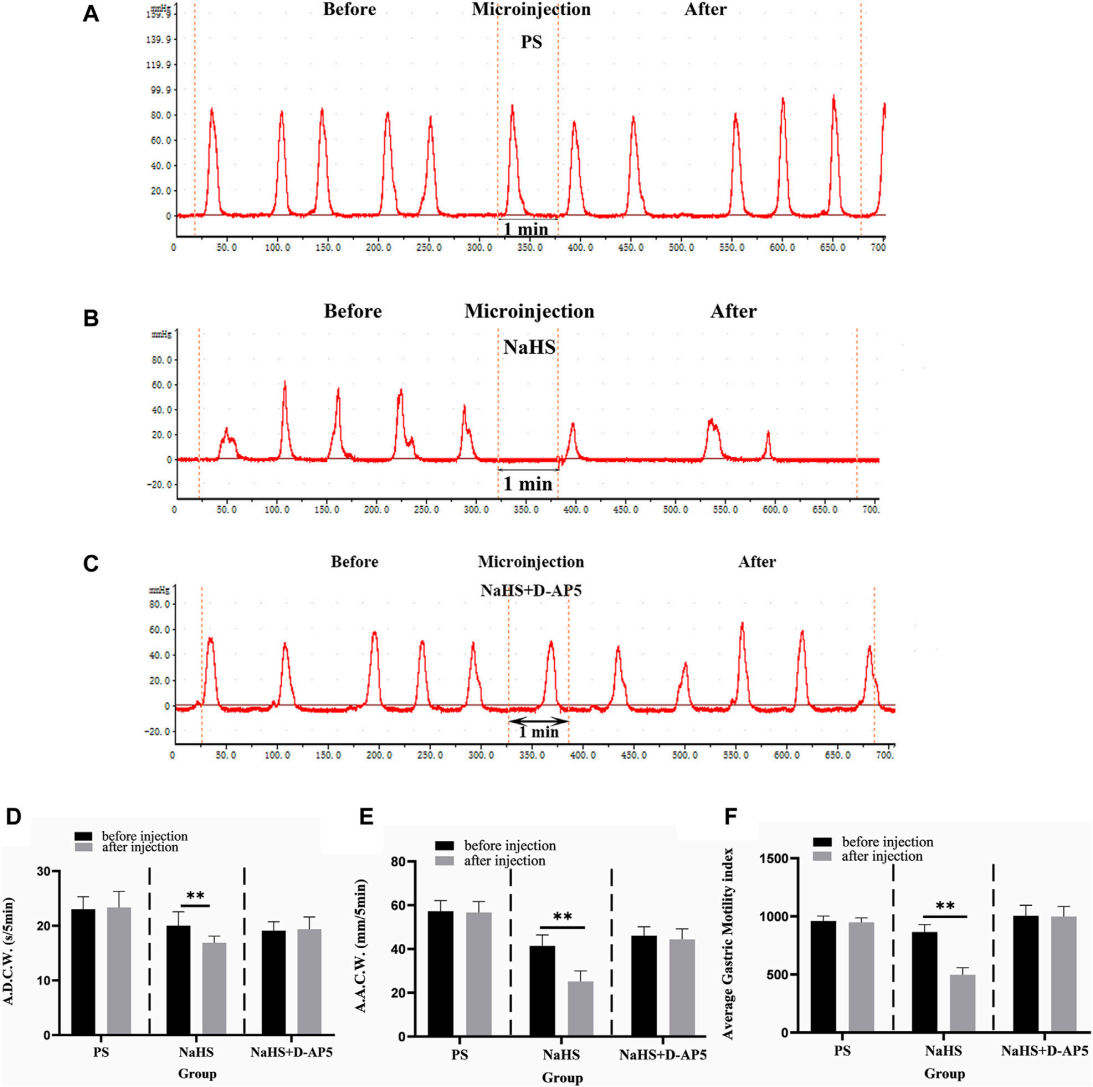
Effect of NaHS microinjection in nucleus ambiguus (NA) on gastric motility in rats. **(A)** The gastric motility curve of physiological saline (PS) group, **(B)** The gastric motility curve of NaHS group, and **(C)** The gastric motility curve of D-2-amino-5-phopho-novalerate (D-AP5) + NaHS **(D)** Average duration of contraction waves (A.D.C.W). data before and after microinjection of PS, NaHS and D-AP5 + NaHS (*n* = 6). **(E)** Average amplitude of contraction waves (A.A.C.W) data before and after microinjection of PS, NaHS and D-AP5 + NaHS (*n* = 6). **(F)** Average gastric motility index (A.G.M.I) data before and after microinjection of PS, NaHS and D-AP5 + NaHS (*n* = 6). **p < 0.01,*p < 0.05, after microinjection compared with before microinjection.

After injecting 2 nmol NaHS into the NA, the images of gastric motility 5 min after drug injection were compared with those that were obtained 5 min before drug injection. The findings showed that the A.D.C.W., A.A.C.W., and A.G.M.I. of gastric motility were inhibited, to some extent, after injection compared with before (Figure 3B). PS and D-AP5+NaHS was injected into the NA, no significant changes in all indicators of gastric motility were observed after the injection, compared with those that were noted before the injection (Figures 3A,C).

The data 5 min before and 5 min after microinjection were also measured, analyzed, and compared. When a 2 nmol NaHS microinjection was administered to the NA, the contraction wave of A.A.C.W decreased from 41.40 ± 2.05 mm 5 min^−1^ to 25.14 ± 1.96 mm 5 min^−1^ (*p* < 0.01); the contraction wave of A.D.C.W decreased from 20.04 ± 1.13 s 5 min^−1^ to 16.89 ± 0.54 s 5 min^−1^ (*p* < 0.01); and A.G.M.I decreased from 865.93 ± 25.91 to 497.37 ± 24.80 (*p* < 0.01) (Figures 3D–F).

Microinjection of D-AP5, followed by NaHS in the NA eliminated the inhibitory effect of NaHS on gastric motility. The data 5 min before and 5 min after the microinjection procedure were measured, analyzed, and compared. The A.A.C.W of the contraction waves changed from 46.11 ± 1.82 mm 5 min^−1^ to 44.35 ± 2.15 mm 5 min^−1^ after D-AP5 injection followed by NaHS injection. The A.D.C.W of the contraction waves changed from 19.12 ± 0.72 s 5 min^−1^ to 19.38 ± 0.99 s 5 min^−1^, and the A.G.M.I changed from 1005.24 ± 40.57 to 998.05 ± 39.09 (Figures 3D–F). These data suggest that NaHS can regulate gastric motility through the NMDA receptors.

### 3.4 Comparison of gastric motility inhibition rate after NA injection drugs in each groups

**FIGURE 4:**
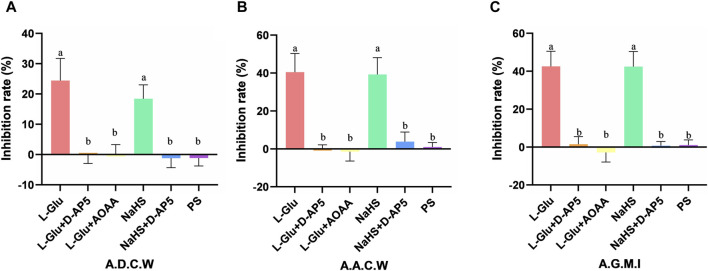
The gastric motility inhibition rate of L-glutamate (L-Glu), D-2-amino-5-phopho-novalerate (D-AP5) + L-Glu, aminooxyacetic acid (AOAA) + L-Glu, NaHS, D-AP5 + NaHS and physiological saline (PS) groups (*n* = 6) **(A)** Average duration of contraction waves (A.D.C.W). inhibition rate in each group. **(B)** Average amplitude of contraction waves (A.A.C.W) inhibition rate in each group. **(C)** Average gastric motility index (A.G.M.I) inhibition rate in each group. Different letters between two groups indicate a significant difference (*p* < 0.05).

The inhibitory rates of A.D.C.W in the L-Glu, L-Glu + D-AP5, L-Glu + AOAA, NaHS, NaHS + D-AP5 and PS groups is 24.43% ± 2.98%, −0.17% ± 1.14%, 0.09% ± 1.30%, 18.42% ± 1.87%, −1.22% ± 1.28% and −1.15% ± 1.08%, respectively (Figure 4A). The inhibition rates of A.A.C.W in the L-Glu, L-Glu + D-AP5, L-Glu + AOAA, NaHS, NaHS + D-AP5 and PS groups is 40.46% ± 4.01%, 0.23% ± 0.78%, −1.48% ± 2.03%, 39.22% ± 3.64%, 3.83% ± 2.05% and 0.94% ± 0.97%, respectively (Figure 4B). The inhibition rates of the A.G.M.I in the L-Glu, L-Glu + D-AP5, L-Glu + AOAA, NaHS, NaHS + D-AP5 and PS groups to 42.56% ± 3.25%, 1.46% ± 1.65%, −2.85% ± 2.07%, 42.40% ± 3.25%, 0.67% ± 0.90% and 1.03% ± 1.11%, respectively (Figure 4C). The data showed that the gastric motility inhibition rate of L-Glu group and NaHS group was significantly higher than that of the blocker groups and the control group. These results indicate that the L-Glu-NMDA receptors-CBS-H_2_S-NMDA receptor pathway in NA regulates gastric motility in rats.

### 3.5 Injecting D-AP5 and AOAA into NA eliminates the promotion of gastric acid secretion by 2 nmol L-Glu in rats

**FIGURE 5:**
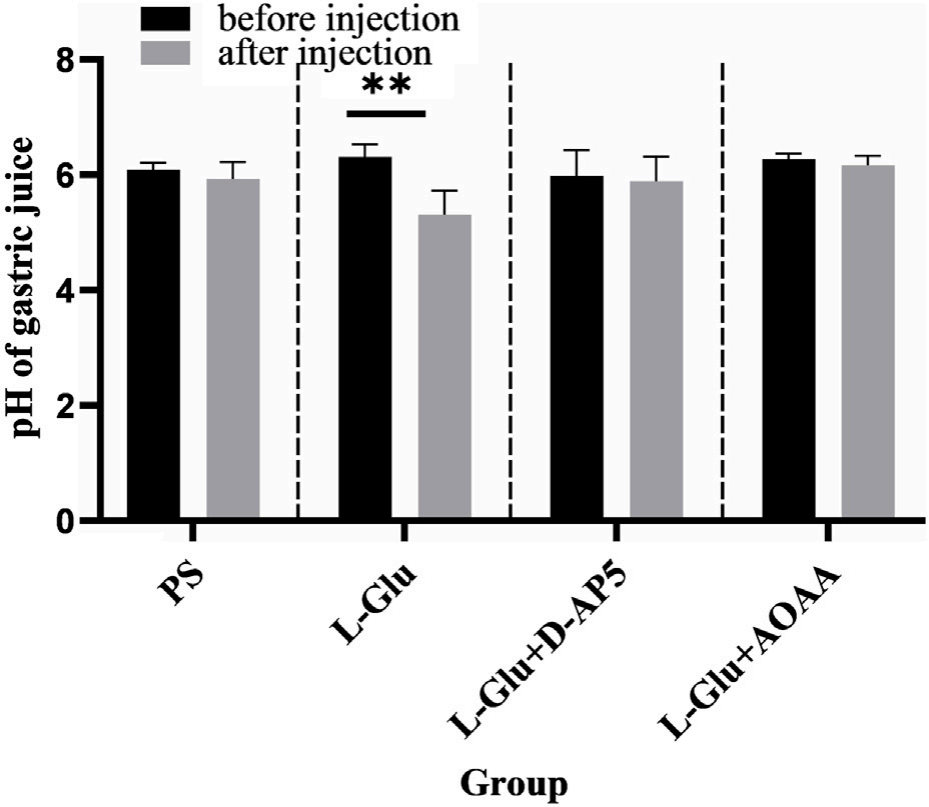
Effects of nucleus ambiguus (NA) injection of physiological saline (PS), L-glutamate (L-Glu), D-2-amino-5-phopho-novalerate (D-AP5) + L-Glu and aminooxyacetic acid (AOAA) + L-Glu on gastric acid secretion (*n* = 6). **p < 0.01, after microinjection compared with before microinjection.

The findings showed that microinjecting 2 nmol L-Glu in the rat NA significantly enhanced gastric acid secretion in rats (Figure 5). No change in gastric acid secretion was noted PS D-AP5 + L-Glu and AOAA + L-Glu was microinjected in rats under the same conditions (Figure 5).

The data 30 min before and 30 min after microinjection were analyzed and compared. The pH of the gastric acid decreased from 6.31 ± 0.09 to 5.31 ± 0.17 (*p* < 0.01) when the NA was injected with a dose of 2 nmol L-Glu. In the control group, the pH of gastric acid reduced from 6.09 ± 0.05 to 5.93 ± 0.12 when NA was injected with PS.

The results from this study showed that when D-AP5, followed by L-Glu, were microinjected in the NA, facilitative effect of L-Glu on gastric acid secretion was eliminated (Figure 5). The data 30 min before and 30 min after the microinjection were analyzed and compared. The pH of gastric acid changed from 5.99 ± 0.18 to 5.89 ± 0.18 after D-AP5 was injected, followed by L-Glu. No significant change in gastric acid pH was observed after microinjecting D-AP5 + L-Glu in the NA. This suggests that L-Glu may regulate gastric acid secretion through the NMDA receptors.

Injecting the NA with AOAA followed by L-Glu eliminated the facilitative effect of L-Glu on gastric acid secretion (Figure 5). The data 30 min before and 30 min after the microinjection were analyzed and compared. The pH of gastric acid changed from 6.27 ± 0.04 to 6.16 ± 0.07 after AOAA, followed by L-Glu injection, was injected. No significant change in gastric acid pH was observed after microinjection of AOAA + L-Glu in the NA. This suggests that L-Glu may regulate gastric acid secretion through the NMDA receptors, followed by CBS neurons.

### 3.6 Injecting D-AP5 into NA eliminates the promotion of gastric acid secretion by 2 nmol NaHS in rats

**FIGURE 6:**
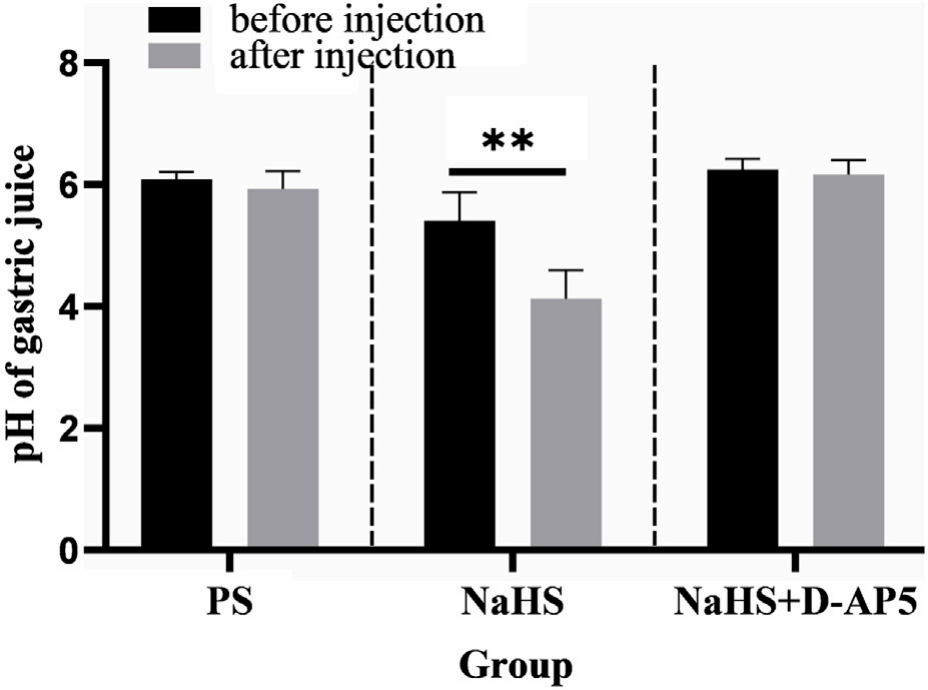
Effects of nucleus ambiguus (NA) injection of physiological saline (PS), NaHS and D-2-amino-5-phopho-novalerate (D-AP5) + NaHS on gastric acid secretion (*n* = 6). **p < 0.01, after microinjection compared with before microinjection.

Injecting 2 nmol NaHS in the rat NA was found to significantly promote gastric acid secretion in rats (Figure 6). Injecting PS under the same conditions did not result in a change in gastric acid secretion in rats (Figure 6).

The data 30 min before and 30 min after the microinjection procedure were analyzed and compared. The pH of the gastric acid decreased from 5.41 ± 0.19 to 4.13 ± 0.19 at when the NA was injected with a dose of 2 nmol NaHS (*p* < 0.01). In the control group, the pH of gastric acid changed from 6.09 ± 0.05 to 5.93 ± 0.12 when PS was injected into the NA.

The findings from this study showed that injecting the NA with D-AP5 followed by NaHS eliminated the facilitative effects of NaHS on gastric acid secretion (Figure 6). The data 30 min before and 30 min after microinjection were analyzed and compared. A change in the pH of the gastric acid from 6.25 ± 0.07 to 6.16 ± 0.10 was noted after D-AP5 injection followed by the NaHS injection. No significant change in gastric acid pH was observed after D-AP5 + NaHS was injected in the NA. This indicated that NaHS may regulate the secretion of gastric acid secretion through the NMDA receptors.

### 3.7 Comparison of gastric acid secretion promotion rate after NA injection drugs in each groups

**FIGURE 7:**
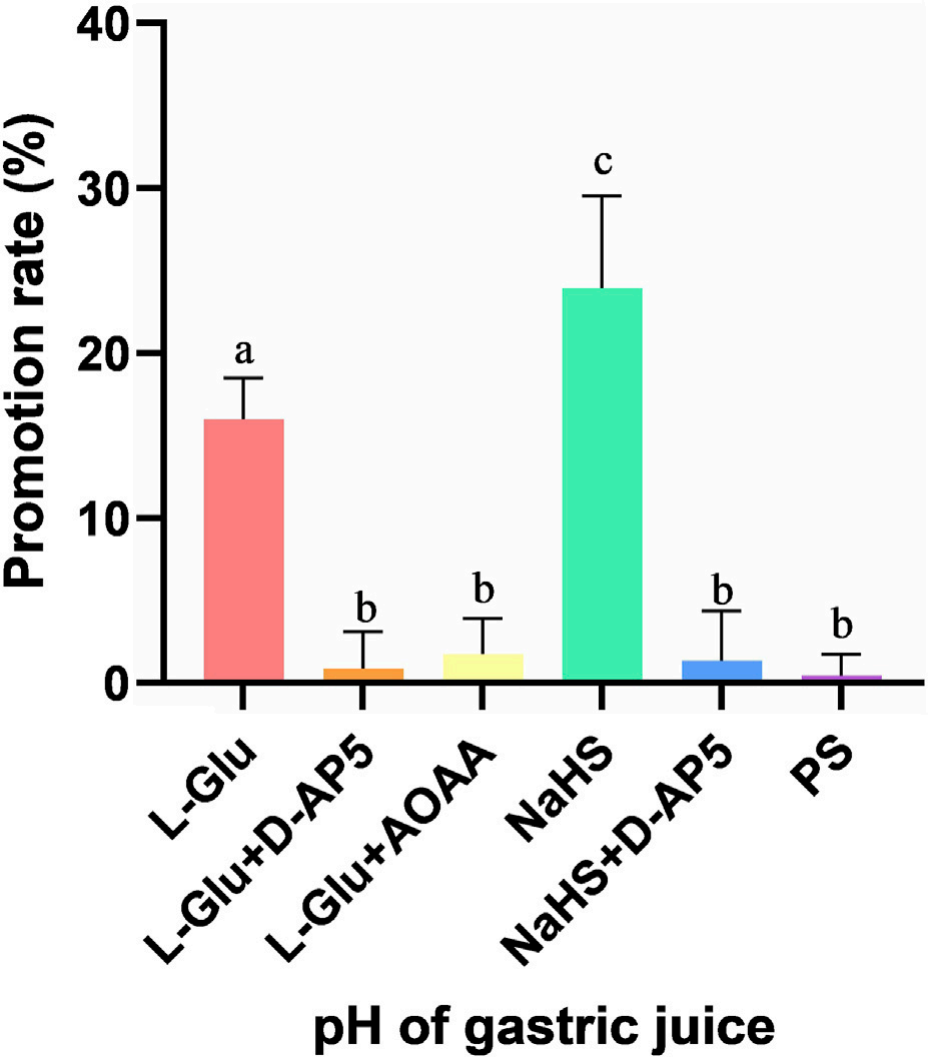
The gastric acid secretion promotion rate of L-glutamate (L-Glu), D-2-amino-5-phopho-novalerate (D-AP5) + L-Glu, aminooxyacetic acid (AOAA) + L-Glu, NaHS, D-AP5 + NaHS and physiological saline (PS) groups (*n* = 6). Different letters between two groups indicate a significant difference (*p* < 0.05).

The promotion rate of gastric acid secretion in the L-Glu, L-Glu + D-AP5, L-Glu + AOAA, NaHS, NaHS + D-AP5 and PS groups is 15.98% ± 1.03%, 0.85% ± 0.92%, 1.74% ± 0.88%, 23.94% ± 2.28%, 1.36% ± 1.23% and 0.44% ± 0.53%, respectively (Figure 7). The data showed that the gastric acid secretion promotion rate of L-Glu group and NaHS group was significantly higher than that of the blocker groups and the control group, and the gastric acid secretion promotion rate of the NaHS group was higher than that of the L-Glu group. These results indicate that the L-Glu-NMDA receptors-CBS-H_2_S-NMDA receptor pathway in NA regulates gastric acid secretion in rats.

## 4 Discussion

The vagal reflex plays an important role in regulating gastric contraction and acid secretion. The vagal afferent fibers transmit sensory information from the stomach to the nucleus tractus solitarius and then to the vagal efferent neurons. The efferent vagal fibers then transmit information with the intramural gastric cholinergic presence Schubert (2007). It’s also important to note that the same vagus nerve that innervates the stomach is present in the NA, suggesting a close connection between the nucleus ambiguus and the stomach Shapiro and Miselis (1985). Electrical stimulation of the NA in rats significantly reduces gastric motility and is blocked by vagotomy Wang et al. (2007). However, it was also found that electrical stimulation of the NA in cats increased the contraction of the gastric sinus and pyloric smooth muscle. It slightly increased the pH of the gastric acid and bicarbonate content, probably due to the different species of animals chosen for the experiment Pagani et al. (1984). This suggests that activation of glutamate receptors in the brain regions that control gastric function may stimulator inhibit gastric secretion, depending on various factors, including the nature of the stimulus, the brain region involved, and the dose of the glutamate receptor agonist or inhibitor used.

H_2_S has been reported to exert anti-apoptotic and anti-inflammatory effects in the nervous system, so its effects on physiological systems such as nerves cannot be ignored Kida and Ichinose (2015). We did a dose-dependent search in previous experiment and found that 2 nmol of NaHS had the greatest effect on gastric function, so we chose 2 nmol Li et al. (2021). Injection of the NMDA receptor blocker, D-AP5, attenuates the gastric mucosal damage caused by restraint water-immersion stress Landeira-Fernandez (2015). However our previous studies have shown that the single injection of NMDA receptor blocker D-AP5 into the ambiguus nucleus has no significant change in gastric motion Sun et al. (2010). H_2_S was also found to enhance the activity of the NMDA receptors activated by glutamate in neurons. Please note that the increased sensitivity of the NMDA receptors was associated with the H_2_S-induced production of cyclic adenosine monophosphate (cAMP) Kimura and communications (2000). Moreover, H_2_S also cascades through adenylate cyclase to modulate the NMDA receptors. It also acts in a dose-dependent manner in appropriate physiological concentrations (10–130 *μ*M) Moshal et al. (2008). We also observed inhibition of gastric motility in the rats after exogenous H_2_S donor NaHS was injected in the NA Sun et al. (2020). Therefore, we suggest that H_2_S can modulate gastric function through the NMDA receptors, but within the normal physiological concentration range.

According to previous literature Shaw et al. (2002), this experiment explored 2 nmol of L-Glu as a chemical stimulant for NA, and the results showed that L-Glu had an effect on gastric function after being injected into NA. Studies have shown that the release of the excitatory neurotransmitter L-Glu from brain neurons increases the production of H_2_S, as a response to neuronal excitation, and activation of glutamate transporters is necessary for exogenous hydrogen sulfide to function He et al. (2017), *in vitro* smooth muscle experiments, CBS inhibitor AOAA significantly inhibited antral smooth muscle contraction Huang et al. (2013). Glutamate is the main excitatory amino acid neurotransmitter in the brain and is involved in various physiological and pathological processes, and plays a role in regulating many functions of the nervous and other systems, including the secretory, motor, and sensory functions of the gastrointestinal tract Filpa et al. (2016), and it is now widely believed that the receptors that regulate vagus excitation are glutamatergic. Blocking the glutamate receptors can prevent endotoxin-induced inhibition of gastric acid secretion García-Zaragozá et al. (2000). In early studies performed on rats, it has been demonstrated that amino acids exert inhibitory effects on tonic and phasic contractile gastric responses by activating the dorsal vagal complex (DVC) Raybould et al. (1989). L-Glu increases the efferent activity of the visceral vagus nerve, leading to enhanced vagal and vagal sympathetic reflexes. Microinjection of L-glutamate into the caudal side of the DMV causes a vagally-mediated depression of gastric motility Zhou et al. (2008). Injecting L-Glu in the lateral hypothalamic region induces increased blood flow to the gastric mucosa, thereby modulating gastric acid secretion Namiki et al. (1993). Our previous study found that L-Glu chemically stimulated DMV and that the NA in the rat medulla oblongata inhibited gastric motility via excitation of preganglionic cholinergic neurons with glutamatergic NMDA receptors, through the NMDA receptor-NO pathway Sun et al. (2010, 2014). We also found that NaHS injection in the NA inhibited gastric motility and promoted gastric acid secretion in rats Sun et al. (2015). This indicated that L-Glu and NaHS exhibit similar modulation of gastric function in the DMV. In the present study, we also found that the injection of L-Glu and NaHS in the NA inhibited gastric motility in rats. This indicated that inhibitory transmitters were released and inhibitory fibers in the vagus nerve were stimulated when the NA was activated. It has also been shown that stimulation of the NTS by glutamate elicits both excitatory and inhibitory DMV neurons, and that activation of glutamatergic neurons ameliorates stress-induced delayed gastric emptying Iwa et al. (2006). Many studies have also shown that glutamate signaling has a role to play in regulating the neuronal connections between the CNS and gut. This constitutes the brain-gut axis Hornby and physiology (2001), along which signals from the CNS to the gut regulate gastrointestinal secretion and motor function, while chemical and electrical signals from the gastrointestinal tract provide sensory information to the CNS. Glutamate is involved in transmitting the sensory input to brain regions involved in regulating different gut functions. This is achieved through activation of the vagal, visceral afferent fibers, and efferent pathways, including the DMV. This drives excitatory and inhibitory inputs to the gastrointestinal tract, which may also be regulated by glutamate receptor activationHornby and physiology (2001). However, further studies are needed to determine the mechanisms through which L-Glu and NaHS regulate gastrointestinal function.

Glutamate is known to act by activating ionotropic ligand-gated cation channels (NMDA, AMPA, and erythropoietin) iGluRs Bleakman and Lodge (1998) and G protein-coupled receptors mGluRs (groups I, II, and III). Previous studies on how glutamate receptors in the CNS contribute to controlling gastrointestinal motility have shown that ionotropic glutamate receptors are involved in regulating the gastrointestinal tract. On the other hand, metabotropic glutamate receptors are not involved in the regulation of the gastrointestinal tract Golovynska et al. (2018). Previous studies strongly suggest that inhibition of gastric motility by NMDA or excitatory amino acids is achieved through central action Shinozaki et al. (1990). Moreover, alterations in NMDA signaling have been shown to occur rapidly in vagal central neural circuits, thereby increasing the vagal efferent drive to the stomach by activating NMDA receptors and upregulating glutamatergic signaling in vagal central neural circuits Clyburn et al. (2018). The NMDA receptors have a functional role in the regulation of autonomic functions of the gastrointestinal tract, including transient lower esophageal sphincter relaxation and gastric acid secretion Sengupta et al. (2004). NMDA receptors may regulate stomach movement through vagal nerve interaction.

According to restraint water-immersion stress studies, excitatory glutamate receptors are present in most neurons. In DVC, activated NMDA receptors can independently cause vagal-mediated gastric motor excitation. Moreover, injection of NMDA in the lateral ventricle increases gastric acid secretion in rats via vagal activation Tsuchiya et al. (2001). NMDA receptors in DVC can independently cause vagally mediated enhancement of gastric motility Sivarao et al. (1999). They also affect vagally mediated gastric function through excitatory glutamatergic synaptic messaging. NMDA in lateral hypothalamus excited most of ghrelin-responsive gastric distension sensitive neurons and promoted gastric motility Gong et al. (2017). Peripheral NMDA receptors are also present in the vagus nerve of the stomach and in primary afferent fibers of the spinal cord. These findings suggest the involvement of NMDA receptors in neurogenic motility, gastric motility Janković et al. (1999), or gastric acid secretion. NMDA receptors play a central role in regulating the transmission of visceral sensitivity information from the gastrointestinal tract to primary neurons in the CNS. Moreover, intrathecal injection of NMDA receptor antagonists into the spinal cord reduces colonic sensitivity to mechanical stimuli, highly suggesting that visceral activity is regulated by NMDA, an endogenous glutamatergic receptor of the mechanosensitive pathway Blackshaw and Gebhart (2002). Stimulating the ST36 acupuncture point enhances gastric motility and is eliminated by the NMDA receptor blocker, D-AP5, but not by AMPA receptor modulation. This supports the findings from an earlier study from our laboratory, which suggested that NMDA receptors play a crucial role in mediating the enhancement of gastric motility Gao et al. (2012). Excitatory amino acids and their receptors may play an important role in the mammalian intestinal system, and gastric ulcers induced by restraint water-immersion stress in mice can be significantly reduced by modulating gastric acid secretion and gastric mucosal blood flow after intravenous administration of L-Glu and NMDA Chen et al. (2001). NMDA glutamatergic receptors are associated with protective molecular mechanisms of the gastric mucosa in animal ulcer models. On the one hand, they are associated with the glutamate-mediated inflammatory effects demonstrated in cultures of intestinal interneurons. The expression of NMDA receptor subunits is not altered in stress-induced ulcer models. This indicates, that NMDA receptors are not directly involved in ulcer formation, but rather in protecting the gastric mucosa Di Cerbo et al. (2020). The current study also revealed that pre-injecting the NMDA receptor blocker, D-AP5, eliminated modulation of gastric function by L-Glu and NaHS.

## 5 Conclusion

In conclusion, we found that microinjection of either L-Glu or H_2_S donor NaHS in the NA inhibited gastric motility. The modulatory effects of L-Glu and NaHS on gastric function were eliminated by pre-microinjection of the CBS inhibitor AOAA followed by L-Glu, and pre-injection of the selective NMDA receptor antagonist D-AP5 followed by L-Glu or NaHS, respectively in the NA. The L-Glu-NMDA receptor-CBS-H_2_S-NMDA receptor pathway in the NA was confirmed to be involved in regulating gastric motility and gastric acid secretion in rats. The corresponding receptor (or channel) blockers or agonists could also be designed later as clinical therapeutic drugs.

## Data Availability

The original contributions presented in the study are included in the article/Supplementary material, further inquiries can be directed to the corresponding author.
